# Magnetic Force-Assisted Nonlinear Three-Dimensional Wideband Energy Harvester Using Magnetostrictive/Piezoelectric Composite Transducers

**DOI:** 10.3390/mi13101633

**Published:** 2022-09-29

**Authors:** Zhiming Lin, Hongyun Li, Shaobo Lv, Binbin Zhang, Zhiyi Wu, Jin Yang

**Affiliations:** 1 School of Electronics and Information Engineering, Southwest University, Chongqing 400715, China; 2 Key Laboratory of Advanced Technologies of Materials (Ministry of Education), School of Materials Science and Engineering, Southwest Jiaotong University, Chengdu 610031, China; 3 Beijing Institute of Nanoenergy and Nanosystems, Chinese Academy of Sciences, Beijing 101400, China; 4 Key Laboratory of Optoelectronic Technology & Systems (Ministry of Education), Department of Optoelectronic Engineering, Chongqing University, Chongqing 400044, China

**Keywords:** energy harvester, magnetoelectric, multi-directional, bandwidth

## Abstract

This paper presents a nonlinear magnetoelectric energy harvester which has the potential to harvest vibrational energy over a wide bandwidth in arbitrary motion directions. Three springs with equal intersection angles are adopted to absorb the multi-directional vibration energy. Magnetic interaction between the magnets and ME transducers allows the nonlinear motion with enhanced harvesting frequency range. Very good agreement is observed between the numerical and experimental open-circuit voltage output frequency response curves. The experimental results show that the harvester can harvest vibrational energy in an arbitrary direction, exhibiting a further bandwidth of 5.2 Hz. This study provides a new solution to effectively use the magnetoelectric energy harvester for multi-directional and bandwidth vibrational energy scavenging in the surrounding environment.

## 1. Introduction

Energy harvesting provides a promising solution to implement self-sustained lower-power electronic devices and which is regarded as a promising alternative to traditional batteries [1]. Vibration to electricity energy conversion strategies have received extensive attention over the past decade. The basic mechanisms of vibration energy harvesting include piezoelectric, refs. [2,3] electromagnetic, refs. [4,5] magnetostrictive, refs. [6,7] and triboelectric transduction [8,9,10]. Nevertheless, many reported devices are not easy to implement in practice due to a number of critical issues, such as diverse mechanical vibrations or varying excitation frequency scenarios, resulting in drastic reduction of the power output. To remedy these key issues of conventional energy harvesters, energy-harvesting systems with three-dimensional (3D) motion directions have been carried out in many researches [11,12,13,14]. Aktakka et al. [11] reported a piezoelectric energy harvester based on transverse-mode piezoelectric crab-leg suspensions and partitioned top electrodes to scavenge ambient vibration energy from all three axes. Liu et al. [12] developed an electromagnetic energy harvester with multiple vibration modes characterized using 3D excitation at different frequencies. However, due to the complicated structure and increased system volumes, power densities and efficiency of energy conversion in such designs are reduced. A tri-directional piezoelectric energy harvester that exhibits the capability of harvesting vibration energy from three orthogonal directions was demonstrated by Su et al. [13] Nevertheless, such energy harvester can only work in a narrow frequency bandwidth due to the ambient vibration with a time-variant frequency.

In this study, we propose a nonlinear magnetoelectric energy harvester which can achieve multi-directional sensitivity and remarkable broad bandwidth. In the proposed energy harvester, three spiral springs at equal central angle with each other make the cylindrical magnet vibrate in arbitrary directions. Magnetic interaction between the magnets is adopted for broadband energy harvesting because of the nonlinearity on the system. Numerical simulations and experimental results are carried out that nonlinear harvester can sustain large-amplitude oscillations over a wide frequency range, and it can generate power efficiently in an arbitrary direction.

## 2. Harvester Design and Analysis Model

**Figure 1:**
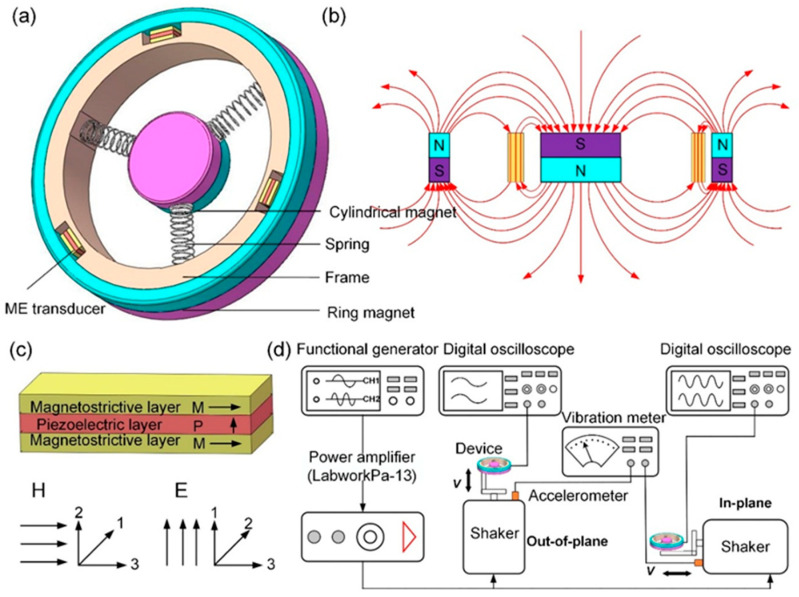
(**a**) Schematic diagram of the 3D wideband magnetoelectric energy harvester. (**b**) Magnetic field produced by the magnetic circuit. (**c**) Operation model of the ME transducer. (**d**) Schematic illustrations of measurement setup.

The novel 3D wideband energy harvester using magnetostrictive/piezoelectric composite (ME) transducers has been designed, which is composed of three identical springs, three ME transducers, a harvester frame, and a magnetic circuit provided by a cylindrical magnet and a ring magnet, as shown in Figure 1a. The cylindrical magnet is mobile and suspended by the three identical springs, resulting in oscillating in three-dimensional space. The magnetic circuit comprises a cylindrical magnet and a ring magnet and this arrangement produces a concentrated magnetic flux gradient through the ME transducers, as demonstrated in Figure 1b. The ME transducer is a sandwich of one piezoelectric ((Pb(Zr_1−x_,Tix)O_3_)) layer bonded between two magnetostrictive layers. The magnetostrictive layers are magnetized along the longitudinal direction (L mode), and the piezoelectric layer is polarized in its thickness direction (T mode). Figure 1c shows the operation model of the ME transducer. ME transducers are fixed symmetrically on the acrylic supporting frame. Owing to an external acceleration from ambient vibration, there will exist a relative motion between the ME transducers and the magnetic circuit. Then ME transducers experience the various magnetic field variations, leading to the stress in the magnetostrictive layers. As a result, the stress is transmitted to the piezoelectric layers, and then generating electrical power due to piezoelectric induction.

**Figure 2:**
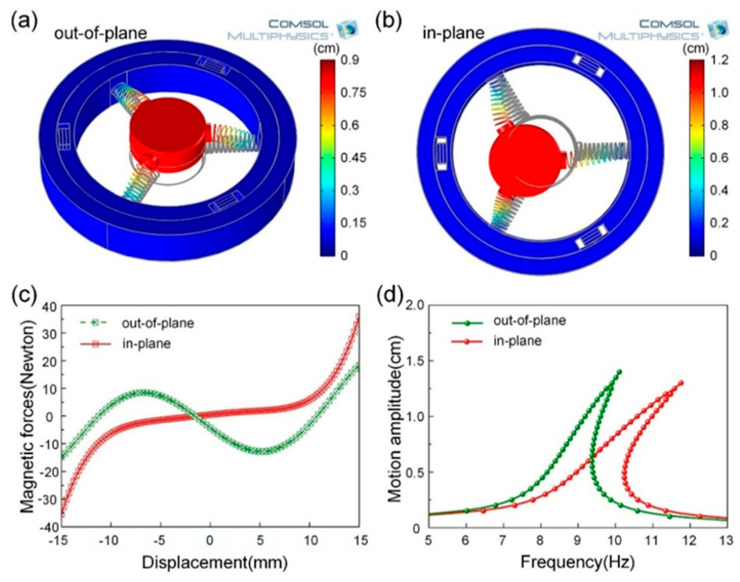
Simulations of the 3D vibration energy harvester. Instant motion state resulting from (**a**) out-of-plane mode, and (**b**) in-plane mode by COMSOL Multiphysics. (**c**) Simulated force-displacement relationship of the cylindrical magnet in out-of-plane mode and in in-plane mode by Ansoft Maxwell. (**d**) Simulated frequency response of out-of-plane mode and in-plane mode with elevated working bandwidth.

In order to design a vibration energy scavenger that meets 3D energy harvesting from diverse mechanical vibrations, theoretically, simulations were conducted by COMSOL Multiphysics in this work. For material data, steel (Young’s modulus of 2.05 GPa and Poisson’s ratio of 0.28) was used for three springs. Note that the magnetic interaction between the ME transducers and magnets is not calculated in the process of simulations in order to simplify the model. The instant motion states of the cylindrical magnetic are shown in Figure 2a,b corresponding to resonant frequencies of 8.4 Hz and 9.2 Hz, respectively, which indicate the mechanical motion behaviors of the harvester under different mode shapes. There are two mode shapes: out-of-plane mode and in-plane mode for the vibration energy harvesting system due to the rationally designed structure. As a result, the proposed harvester is capable of extracting vibration energy from arbitrary directions.

As mentioned above, magnetic interaction between the ME transducers and the magnets exists in the vibration energy harvesting system. Thus, the motion behaviors of the cylindrical magnet in an arbitrary excitation direction will be affected by the magnetic force. The proposed 3D energy harvester could be modeled as the mechanical spring mass-damper system to analyze the frequency response in out-of-plane mode (note that the harvester excited in in-plane mode shares the same model), the governing dynamic equation of motion for this system can be given by

(1) $$m\times \ddot{x}+c\times \dot{x}+k\times x=-m\times \ddot{a}+F\left(x\right)$$

where *m* is the mass of the cylindrical magnet, *c* and *k* are the mechanical damping coefficient and spring constant of the spring, respectively. *x* is the relative displacement of the cylindrical magnet to the acrylic supporting frame, $\dot{x}$ and $\ddot{x}$ are the relative velocity and acceleration of the cylindrical magnet with respect to the frame. $\ddot{a}$ is the acceleration of the supporting frame along the z-axis direction. F(x) denotes the vertical component of the magnetic forces between the ME transducers, which is expressed as a summation of the magnetic forces acting on the cylindrical magnet by the ring magnet, $F_{\mathrm{mag}}\left(x\right)$, and the mounted ME transducers,$\;F_{\mathrm{ME}}\left(x\right)$, i.e.,

(2) $$F\left(x\right)=F_{\mathrm{mag}}\left(x\right)+F_{\mathrm{ME}}\left(x\right)$$

To investigate the influence of F(x) on the motion behaviors of the cylindrical magnet, Ansoft Maxwell 3D software was used as in further study of the relationship between magnetic force and displacement, as demonstrated in Figure 2c. It is clear that the magnetic forces are nonlinear functions of displacement and which are different from each other. It is reasonable to simplify the function of magnetic force into a third-order polynomial and can be written as $F\left(u\right)=k_{l}x+k_{n}x^{3}$(see Reference [15] for details), where the coefficients $k_{l}$ and $k_{n}$ are the fitting parameters, which can be calculated by using a least square procedure. Then, the nonlinear factor related to the motion of the cylindrical magnet evaluated as

(3) $$\alpha =k_{n}\times Y^{2}/k-k_{l}$$

where $Y^{2}=mg/k$ [16]. The working bandwidth of harvester is determined by the nonlinear factor, with a larger value resulting in a wider bandwidth. To further investigate the nonlinear behaviors of the magnetic on the bandwidth, Equation (1) can be given in non-dimensional form as

(4) $$\ddot{x}+2\xi \omega_{0}\dot{x}+\omega_{0}^{2}\left(x+\varepsilon x^{3}\right)=A\omega_{0}^{2}\cos\left(\omega t+\theta \right)$$

where ξ is the damping ratio, *ε* is the third order coefficient of the magnetic force expansion. $\omega_{0}$ is the natural frequency, A is motion amplitude of the acrylic supporting frame, ω and θ are the angular frequency and the phase angle, respectively. Using the Harmonic Balance method (x=Xcosωt is assumed), Equation (4) can be solved. The frequency-amplitude relationship for the harvester can be predicted by

(5) $$\gamma ={\left[1+\frac{3\varepsilon }{4}X^{2}-2\xi^{2}\pm {\left\{{\left(\frac{A}{X}\right)}^{2}-4\xi^{2}\left(1+\frac{3\varepsilon }{4}X^{2}-\xi^{2}\right)\right\}}^{\frac{1}{2}}\right]}^{\frac{1}{2}}$$

where $\gamma =\omega /\omega_{0}$. Subsequently, the typical frequency-response curves (FRC) for a hardening system are plotted using Equations (5) in out-of-plane and in-plane modes, respectively, as depicted in Figure 2d. An intensely nonlinear behavior appears in each FRC with the magnetic force, which contributes the harvester to operating in a wider bandwidth. Thus, the amplitude of frequency-response jumps to a higher level due to the nonlinear behavior of the cylindrical magnetic, leading to a wider resonance range and a higher resonance drop point.

**Figure 3:**
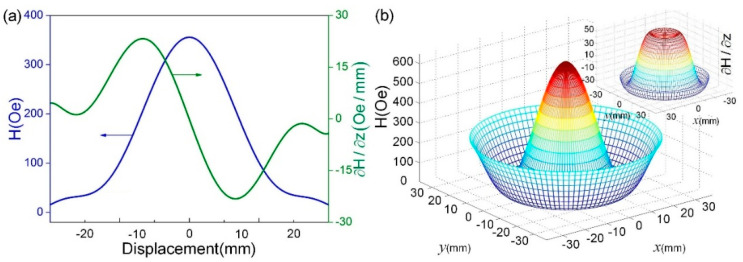
Simulated magnetic fields and variations: (**a**) along z-axial, and (**b**) in-plane mode.

Owing to the motion of the cylindrical magnetic, the fixed ME transducer undergoes a changing magnetic field, then electrical power will be generated. The ME output voltage can be written as

(6) $$V_{\mathrm{out}}=\Delta \bar{H}\times \alpha_{\mathrm{ME}}$$

where $\Delta \bar{H}$ is the magnetic field variation induced by the ME transducer, $\alpha_{\mathrm{ME}}$ is the ME voltage coefficient under a DC magnetic bias field along longitudinal direction. In order to investigate the influence of the magnetic field distribution on the ME voltage output, Ansoft Maxwell 3D software was used to elevate the distributions of magnetic field and variations of the magnetic circuit, as illustrated in Figure 3. Figure 3a,b show that, in the range of −10 mm to 10 mm, the average magnetic fields provided by magnetic circuit are ~230.7 Oe and ~410.2 Oe for the two modes, and the one in in-plane mode is close to the measured optimal bias magnetic field of the ME transducer, ~405 Oe [17]. By contrast, the magnetic field variation in in-plane mode is larger compared with the magnetic field variation in out-of-plane mode. In this case, the output voltage would be larger in in-plane mode, because the ME transducer not only works in a good condition of the DC bias magnetic field but also experiences a large enough magnetic field variation.

## 3. Fabrication

The fabrication process of the designed energy harvester using magnetostrictive/piezoelectric composite transducers was composed of three parts, ME transducers, energy harvester frame and a magnetic circuit. First, three ME transdusers were fabricated by piezoelectric and magnetostrictive layers, one piezoelectric layer is bonded between two magnetostrictive layers using insulated epoxy adhesive, and both the dimensions of the piezoelectric and magnetostrictive layers are 12 mm × 6 mm × 1 mm, being 12 mm in the longitudinal direction. An acrylic sheet was cut into the desired ring shape by a laser cutter (PLS6.75) with the inner and outer diameters of 54 mm and 64 mm, respectively. Three grooves with an included angle of 120° between each other were cut on the outside of the ring acrylic, forming the harvester frame. Then a cylindrical magnet with diameter of 20 mm and height of 8 mm was fixed by three spiral springs with equal intersection angles. And a ring magnet with the inner and outer diameters of 64 mm and 74 mm, was mounted on the outside acrylic frame. Finally, Three ME transducers were mounted symmetrically on the prepared grooves of the acrylic frame. And the wires was led out of the ME transducers to the experimental test system.

## 4. Experimental Results and Discussion

**Figure 4:**
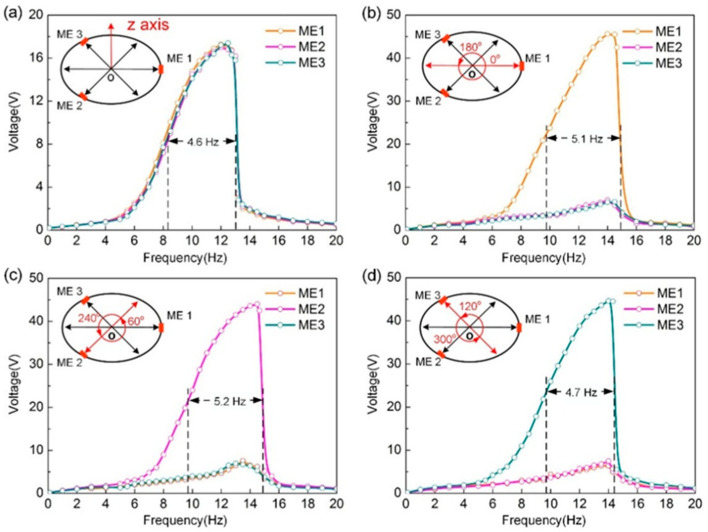
(**a**) The measured open circuit voltages versus frequency with an input acceleration of 1 g in out-of-plane mode. The measured open circuit voltages as a function of frequency at excitation angles of (**b**) 0° (180°), (**c**) 60° (240°), and (**d**) 120° (300°) in in-plane mode.

To measure the output characteristics of the prototype, experimental platform was established, Figure 1d shows the schematic illustrations of experimental test system clearly. We measured the open-circuit voltages for all three ME transducers in out-of-plane mode, as shown in Figure 4a. The voltage outputs of those follow a similar trend and are fairly close to each other. The reason is that three ME transduces experience a uniform magnetic field variation. Want’s more, hardening system responses of the harvester can be observed due to the nonlinear behavior of the magnetic force applied to the movable magnet. As a result, the working bandwidth was broadened, which is well match with the model predictions shown in Figure 2d. If the half peak voltage point is adopted as the criteria of the working bandwidth [18], an approximate working bandwidth of 4.6 Hz can be achieved.

For the capability of vibration energy harvesting in in-plane mode, the experimental voltage responses of the harvester at different excitation angles are measured, as displayed in Figure 4b−d, respectively. It can be found that the proposed harvester could scavenge and convert mechanical vibrations into electricity at different excitation angles, and the experimental results could be extended to the full range of 360° because of the structural symmetry. Besides, one ME transducer with larger output voltage at different excitation angles can be observed, owing to the difference of magnetic field variations. Moreover, the nonlinear behavior of the energy harvesting system leads to broadening the working bandwidth and the maximum working bandwidth can get up to 5.2 Hz, which is well consistent with the simulated results in Figure 2d and theoretical analysis.

**Figure 5:**
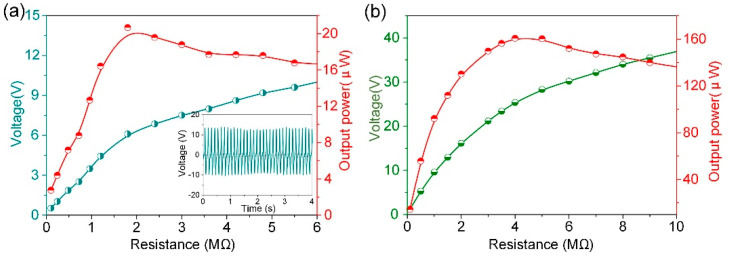
(**a**) The measured output voltage and power of a ME transducer versus the changing load resistance in out-of-plane mode, insert is the real time voltage response. (**b**) The output voltage and power of ME transducer versus the load resistance at the excitation angle of 60° in in-plane mode.

The electrical output power of the harvester was further characterized at the driving frequency of 12 Hz in out-of-plane mode and 14 Hz in in-plane mode, external load was connected to the harvester in series, and the output voltage and power across the resistor were measured, as shown in Figure 5. As the increase of the resistor, the output power calculated by *P* = *V^2^⁄R* will increase dramatically and then decrease slowly. The peak power arrives at the maximum value of 20.7 μW at 1.8 MΩ in out-of-plane mode, while the corresponding maximum output power density of 8.24 μW/cm^3^ can be obtained (Figure 5a). Due to the structural symmetry of the harvester working at in in-plane mode, the experiments of the output power at the excitation angle of 60° was employed, as demonstrated in Figure 5b. The measured maximum output power reaches 160.8 μW with the matched resistance of 4 MΩ. And the corresponding output power in in-plane mode at its matched resistance gets up to 64 μW/cm^3^. Therefore, the above experimental results reveal the output performance of the proposed device for vibrational emery harvesting.

## 5. Conclusions

In summary, we proposed a novel design of three dimensional vibration energy harvester with a broad operating frequency range. The theoretical analysis and experimental characterization are conducted for the proposed harvester. By mean of unique structural design, the harvester can scavenge ambient vibration energy in arbitrary directions. Assisted with the nonlinear behaviors of the movable magnet, the operating bandwidth of the harvester was shown to be substantially enhanced. With further optimization of the dimensional parameters, we believe the proposed design could be promising for practical applications to vibration energy scavenging from the environments for realizing the self-powered wireless sensor networks.

## Data Availability

The data presented in this study are available upon request from the corresponding author.
